# Enhancing nurses’ sustainability consciousness and its effect on green behavior intention and green advocacy: quasi-experimental study

**DOI:** 10.1186/s12912-025-02987-0

**Published:** 2025-04-30

**Authors:** Ahmed Abdellah Othman, Hanan Abdelrazik Abdelall, Hind Ismail Ali

**Affiliations:** 1 Department of Nursing Administration, Faculty of Nursing, Sohag University, Sohag City, Egypt; 2 Department of Nursing Sciences, Collage of Medical Applied Sciences in Wadi El Dawasir, Prince Sattam Bin Abdul Aziz University, Wadi Addawasir, Kingdom of Saudi Arabia; 3 Department of Medical-Surgical Nursing, Faculty of Nursing, Assuit University, Assuit City, Egypt

**Keywords:** Green advocacy, Green behavior intention, Quasi-experimental, Sustainability consciousness

## Abstract

**Background:**

Unless prompt measures are taken, healthcare will continue to exacerbate climate change and its effects while also driving up global demand for care. Nurses are in an optimal position to take on sustainability initiatives because they serve as the main healthcare providers. Due to this, nurses need to be ready for their role in sustainable development. This study aimed to enhance nurses’ sustainability consciousness and assess the effect of that on their green behavior intention and green advocacy and assesses the mediating role of green behavior intention between sustainability consciousness and green advocacy.

**Methods:**

A quasi-experimental research design was adopted to examine the effect of researcher intervention and to investigate causal relationships among study variables at Sohag University Hospitals The study sample consisted of 114 nurses who were invited to be recruited voluntarily in the study. Data were collected using three scales that involved a sustainability consciousness scale, a green behavioral intention scale, and a green advocacy scale. The collected data was analyzed by SPSS 26.0 using number and percent (N %), mean and standard deviation (mean, SD), independent t-test, paired t-test, and PROCESS macro technique, and two groups were formulated.??

**Results:**

A statistically significant difference in the mean scores of sustainability consciousness (103.76 ± 21.81 VS 71.63 ± 22.31), green advocacy (11.50 ± 2.057 VS 8.16 ± 2.37), and green behavioral intention (12.315 ± 2.366 VS 7.08 ± 2.665) was observed between participants nurses in both the intervention and control group (*P* = 0.000).

**Conclusion:**

Enhancing nurses’ sustainability consciousness improves their green behavior intention and green advocacy. Green behavior intention plays a strong mediator role between sustainability consciousness and green advocacy. The study proves that the adoption of sustainability consciousness (SC) will raise a generation of nurses who are sufficiently aware of environmentally sustainable actions. Along the same line, Hospital managers should set strategies to raise healthcare providers’ awareness of sustainability issues and environmentally friendly practices.

## Background

Health and well-being are influenced by the environment, which can have a detrimental effect if it is poisonous or out of balance [1]. Air, water, and soil pollution, along with exposure to toxic chemicals, pose significant health threats. The World Health Organization estimates that Between 2030 and 2050, climate change is expected to cause approximately 250,000 additional deaths per year, from malnutrition, malaria, diarrhea, and heat stress alone, highlighting the direct relationship between human health and the environment. This underscores the urgency of addressing these challenges comprehensively [2].

Rising costs of living, unprecedented weather patterns, and global pandemics are just some of the many issues causing an increased burden on health. Thus, it is essential to integrate sustainable methods into all aspects of human efforts [3]. The need to transition to more sustainable conscious practices is now recognized by the healthcare sector. The energy needed to run medical equipment, pharmaceutical waste, and hospitals’ carbon footprint are a few of the problems that call for smart green solutions [4]. Sustainability in the healthcare industry refers to the ability to deliver services over time while taking future generations into consideration [5].

Sustainability cannot be conceived as a fixed, sequential process or as an anticipated outcome. Instead, it is a multifaceted process that necessitates understanding and analysis on numerous fronts [6]. Managers must comprehend these interrelated concepts of sustainability to accomplish sustainable development in any organization. A person’s awareness of the environment and their experience or knowledge of sustainable facts and situations, including their thoughts, feelings, and behaviors, are referred to as sustainability consciousness [7]. Sustainability knowledge will influence the attitude and behavior of persons. Sustainability knowledge also improves people’s ability to deal with environmental and development concerns [8]. The three dimensions—the environment, society, and economy—were investigated by SC [9].

Sustainability behavior can be characterized as an attitude- and knowledge-driven cognitive process. Moreover, a person’s belief system affects their conduct [10]. People and organizations that care about sustainability are more inclined to embrace green practices because they understand the gravity and consequences of environmental issues [11, 12]. To promote sustainable changes, nurses had to include various green practices in their daily work that adhered to sustainable ideals [13]. Recently, the term “green” has come to refer to the use of environmentally sensitive products and services [14].

Researchers describe the term “employee green behavior” as a set of activities taken by staff members in the workplace to sustain the environment and promote the organization’s sustainable growth [15]. These green behaviors reflected in many nurses’ practices, such as turning off the lights before leaving the room, updating a file electronically, holding meetings by teleconference, or printing drafts on waste paper, are indispensable [16]. According to the International Council of Nurses, nurses must work together to preserve and guard against environmental deterioration, pollution, depletion, and destruction [17].

The level of green behavior is affected by their intention levels. Based on the theory of planned behavior (TPB), individual behavior is driven by behavioral intentions. Behavioral intentions are a function of attitudes specifically about that behavior, related to the performance of that behavior, and the individual’s perception of his/her ability to perform or change the behavior [18]. Previous studies mentioned the concept of green behavioral intention as effective and purposeful acts designed to conserve the physical microenvironment for future generations and neighboring areas [19].

According to Ajzen (1991) [20], intention towards a behavior is a sentiment, either positive or negative, towards an objective object or the performance of a certain behavior. The stronger the conduct, the more favorable the intention toward an action. The intention to engage in green behavior influences nurses’ green behavior positively [21]. According to Leu (2021) [22], Conscious nurses were able to develop a constant green behavior as value orientation as the primary conditioning component for repeated green activities. Green practices can guarantee that resources and goods utilized in healthcare operations are sourced ethically [23].

Nevertheless, despite its importance to employee green habits and the environmental sustainability of firms, employee green advocacy has received little attention [24]. According to Roy (2023) [14] green advocacy refers to openly discussing environmental sustainability, sharing relevant knowledge, and communicating various views in order to encourage others to engage in eco-friendly behavior and consider it as a type of voluntary green activity also, contends that mandatory green behavior among employees is favorably correlated with green advocacy, based on the self-perception theory.

Successful managers support long-term green behavior intention through developing green advocacy. Employee green advocacy is characterized by the positive actions organizational members take to encourage others to behave environmentally. According to cognitive consistency theory Korman, (1970) [25], individuals tend to engage in behaviors consistent with their self-cognition to maintain a consistent evaluation of self-image and reduce possible cognitive disorders. Individuals tend to perform certain behaviors to keep their cognition consistent, so when the employees feel accountable for the environment, they are motivated to engage in green advocacy [26].

Nurses have a responsibility to promote sustainability in healthcare organizations and to advance nursing competencies connected to environmental sustainability [27]. Even though the idea of advocacy seems appealing conceptually, relatively little research has been done on the structures that lead to green advocacy. Furthermore, nurse behavior can be effectively encouraged by education and training programs, but interventions should also address the obstacles that nurses encounter when attempting to adopt sustainable behaviors [28]. Consequently, it is critical to raise nurses’ awareness of sustainability, green advocacy, and green behavioral intention.

Sustainability is acknowledged as a valuable area of quality in Egypt’s healthcare system by Vision 2030. Many writers have emphasized how important nursing is to reaching the Sustainable Development Goals, particularly Goal 3, and how crucial nursing education is to raising environmental consciousness, which is the focus of Goal 4 [29]. Sustainable healthcare methods lower their negative effects on the environment, encourage cost savings, improve patient care, and boost the standing of healthcare professionals. People who receive educational sessions on sustainability and green practices are better able to see things from a systems perspective, comprehend how social, environmental, and economic aspects interact, and make decisions that will benefit future generations [30]. Therefore, developing educational sessions to enhance nurses’ sustainability consciousness, is crucial to improve the green behavioral intents of nurses, which is necessary to understand their willingness and commitment to participate in sustainable and green advocacy.

Therefore, developing educational sessions to enhance nurses’ sustainability consciousness is crucial to improve the green behavioral intents of nurses, which is necessary to understand their willingness and commitment to participate in sustainable and green advocacy.

### Aim of the study

This study aimed to enhance nurses’ sustainability consciousness and assess the effect of that on their green behavior intention and green advocacy. Also, assesses the mediating role of green behavior intention between sustainability consciousness and green advocacy.

### Research hypotheses

#### H1

Sustainability consciousness educational programs improve nurses’ green behavior intention and green advocacy positively.

#### H2

**Fig. 1:**
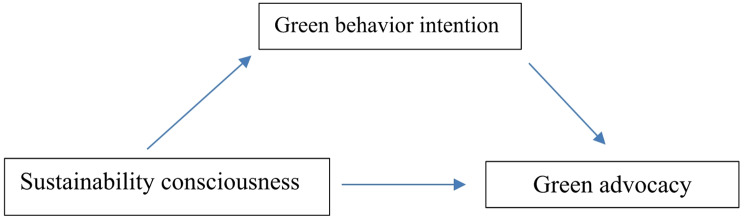
Conceptual framework of the study variables

Green behavior intention has a mediating role between sustainability consciousness and green advocacy (Fig. 1).

## Methods

### Study design

Pre-posttest- a quasi-experimental design was used to carry out this study in which the dependent variables were assessed in one participant group both before and after an intervention. At the same time, the same dependent variables were assessed at pretest and posttest in a different group.

### Participants and setting

The study was carried out at Sohag University Hospital: it is a multi-specialty governmental hospital that consists of six buildings, Each building has five floors and 913 beds, with a range of staff nurse specialties including diploma, technical, and baccalaureate nurses. In the previously indicated settings, 114 nurses participated in this study as participant nurses.

Participants in the study were split into two groups: the intervention group (*n* = 57) and the control group (*n* = 57). Researcher to be sure that the sample size was adequate G*Power software was used to calculate the study’s sample size as a power analysis is most often run before data collection so that the researcher can determine the minimum sample size needed to have enough power to detect an effect [31] So the difference between independent means (two groups) statistical test was chosen by the researcher, and the sample size was determined using an effect size of dz = 0.5. An intervention group (*n* = 57) and control groups (*n* = 57) with a sample size of 114 people were needed to complete the study for it to be powered at 75%, alpha = 0.05, and allocation ratio N2/*N* = 1 based on the previous study used same effect size 0.5 [32].

In order to avoid selection bias, the research converted the nurses’ names to numbers before randomly assigning participants to the study groups using the Spss program, select case, and random sample of cases method. The computer determined which participants were assigned to each group in a randomized manner without the need for human intervention. Additionally, this process was used to select 114 nurses from among the hospital’s available nurses. Pre-posttest, a quasi-experimental approach, was used to carry out the investigation.

### Study instruments

Tool I: It is divided into two sections:

Section 1: Personal data: This section contained the age, gender, marital status, level of education, and prior training in sustainability and green practices of the nurses who were involved.

Section 2: Sustainability Consciousness Scale: Gericke, et al. (2019) [7] Adopted it using the UNESCO definition of sustainable development as a basis. It was used to gauge the behavior, attitudes, and knowledge of nurses on sustainable development. There were 27 statements total, broken down into three categories: sustainability behavior (9 things), sustainability attitudes (9 items), and sustainability knowledge (9 items). The sustainability consciousness questionnaire’s five-point scoring system was applied. The Likert scale included five points: 1 (Strongly disagree), 2 (disagree), 3 (neutral), 4 (agree), and 5 (strongly agree).

According to Gericke et al. (2019) [7], sustainability consciousness is a viable and dependable tool. The Cronbach’s alpha coefficients for sustainability behaviors (α = 0.72, sustainability attitudes (α = 0.78), and sustainability knowingness (α = 0.70) were reported.

Tool II: Green Advocacy Scale: Using three items, it was modified by Kim et al. (2017) [33] To gauge green advocacy. Participants were specifically asked to assess the extent to which they inform and persuade other staff members to work in a hospital setting in a friendly manner. “I try to convince my group members to reduce, reuse, and recycle office supplies in the workplace” is one example from it. On 5-point ratings, where 1 represents strongly disagree and 5 represents strongly agree, the nurses gave their responses. The Cronbach’s alpha score of 0.84 indicates that the three-item scale has adequate internal consistency [33].

Tool III: Green Behavioral Intention Scale: To gauge the participants’ daily intents for green behavior, the researchers used the behavioral intention scale from Norton et al. (2017) [34], which was created based on Ajzen’s planned behavior theory. It had three items on it, like “I intend to perform pro-environmental behaviors while at work tomorrow.” On 5-point measures that went from 1 (strongly disagree) to 5 (strongly agree), participants indicated their answers. For this scale, the Cronbach’s alpha was 0.840 [34]and 0.848 [35].

Validity and reliability: Three nursing administration specialists validated the research tools. Validity for both content and construct was attained. When creating the instrument’s final version, all of the experts’ suggestions for improvements and constructive criticism were taken into account. The researchers used reliability to examine the instruments’ internal consistency; to quantify the measurement consistency of these instruments, reliability was assessed. It was acquired by figuring out each scale’s correlation coefficient. The sustainability consciousness scale for nurses had a Cronbach’s alpha coefficient of 0.82. The Green Behavioral Intention scale was 79 and the Green Advocacy scale was 86.

Pilot study: Before the start of data collection, a pilot study was carried out. The study involved 14 nurses, or 10% of the total study sample, who were recruited to assess the tools’ objectivity, clarity, feasibility, and application as well as to estimate the time required for data collection. Items were corrected, modified, omitted, and added as needed in accordance with the data analysis results. Because of the small change, a pilot study was included in the study sample.

Ethics consideration: The researcher received formal approval to conduct the current study from the Sohag University Hospital administrator and the ethics committee with the code (N.168). All research participants gave their informed consent after being made aware of the study’s goal to win their cooperation and trust. Every participant was given the option to either stay in the study or stop. Confidentiality and privacy were guaranteed. Respect was shown for ethics, morals, culture, and beliefs.

### Study procedures

The study procedures were conducted over three phases: preparatory, implementation, and evaluation. Three stages comprised the study procedures: planning, carrying out, and assessing. The study phases were finished in four months, starting in April 2024 and ending in July 2024. Two times during the study, the data were gathered using various tools: once before the program began and again after a month had passed since it began. The research was carried out in the following three stages:

Phase of preparation: The study design and data collection tools are based on a review of previous, current, and international literature about the detrimental effects of climate change on health. This is done through the use of computer searches, periodicals, magazines, and books to compare the study’s data collection tools and create a green education program. The director of Sohag University Hospital received an official letter of authorization from the dean of the nursing faculty at Sohag University.

Phase of implementation: This study’s implementation phase ran from the start of May 2024 until the end of June 2024. Sample for study: 114 nurses were split into a control group (*n* = 57) and an intervention group (*n* = 57). As a baseline data set (pretest), the control and intervention groups fill out the questionnaire. Only the intervention group (*n* = 57) received instructional program sessions from the researcher. To stop the nurses from dropping out of the units, the intervention group split into two subgroups (A&B): group A (*n* = 29) and group B (*n* = 28). The head nurses of departments and the nursing director of Sohag University Hospitals coordinated the program’s educational session attendance. A two-hour instructional program delivered in four sessions per week, lasting two hours each. The course material provided fundamental information about sustainable healthcare practices and their significance for both individual and global health. Additionally, the nurse’s contribution to green practices and their pro-environmental and eco-friendly activities. Researchers employed a range of instructional strategies, including brainstorming, group discussions, and lectures. Furthermore using other audiovisual tools, such as exchanging images, posters, and videos.

The phase of evaluation: The same study evaluation instruments, it was utilized to evaluate participants’ sustainability consciousness, green advocacy, and green behavioral intention before and after the implementation of educational programs in order to identify any changes, similarities, and potential improvement areas.

Evaluation phase: Using the same study assessment instruments, participants’ sustainability consciousness, green advocacy, and green behavioral intention were evaluated before and after the educational program was implemented. This allowed for the identification of differences, similarities, areas for improvement, and flaws. After a month of program implementation, the researcher conducted a post-program evaluation using the same instruments to confirm whether or not the participating nurses had truly reported improvements in the three variables.

### Data analysis

Utilizing SPSS 26.0 (IBM Inc., Chicago, IL, USA) for data analysis, the responses from the 114 nurses who were recruited were examined. While continuous variables were characterized by the mean and standard deviation (mean, SD), categorical variables were described by number and percent (N %). Additionally, the differences between the pretest and post-test were evaluated using the Independent t-test. The difference between the intervention and control groups was assessed using a paired t-test. Lastly, the mediation model was examined using SPSS’s PROCESS macro approach from the regression analysis section to determine mediation analysis. When the P-value was less than 0.05, it was deemed statistically significant, and when it was less than 0.001, it was deemed statistically extremely significant.

## Results

Table 1: Shows that, less than half of the study participants in the intervention and control group (45.7% & 47.4%) were aged between 25 < and 30 years with Mean ± SD 33.02 ± 5.931, (71.9% & 77.2%) female, (66.7% & 61.44%) married, (42.1% & 49.1%) had Associate degree in nursing and (87.7% & 84.2%) of them hadn’t previous training or education related to sustainability and green behavior. Also, there was no significant difference between the intervention and control groups regarding their data.

**Table 1:** Comparison of the participant nurses’ characteristics (*n* = 114)

Demographic data	Intervention(57)		Control(57)		*P*
	No	%	No	%	
**Age**					2.65 0.492
< 25 years	14	24.5	12	21.1	
25 < 30 years	26	45.7	27	47.4	
30 < 40 years	14	24.5	14	24.5	
≥ 40 years	3	5.3	4	7.0	
**Mean ± SD** 33.02 ± 5.931					
**Gender**					1.84 0.371
Male	16	28.1	13	22.8	
Female	41	71.9	44	77.2	
**Marital status**					2.945 0.104
Single	11	19.3	15	26.3	
Married	38	66.7	35	61.4	
Divorced	6	10.5	6	10.5	
Widow	2	3.5	1	1.8	
**Educational preparation**					0.947 0.235
Diploma in Nursing	12	21.1	9	15.8	
Associate degree in nursing	24	42.1	28	49.1	
Bachelor’s degree in nursing	20	35.0	18	31.6	
Postgraduate education	1	1.8	2	3.5	
**Previous training/education related to sustainability and green behavioral**					2.03 0.341
Yes	7	12.3	9	15.8	
No	50	87.7	48	84.2	

Table 2: shows the level of sustainability consciousness and its subscales: sustainability knowingness, sustainability attitudes, and sustainability behavior. At the beginning of the study, the mean scores of sustainability consciousness in the intervention and control groups of pre-program were (68.35 ± 14.55 and 70.01 ± 13.34), respectively, showing no significant difference between the two groups at (*P* = 0.20). The mean scores of sustainability consciousness of the intervention and control groups of post-program were (103.76 ± 21.81 and 71.63 ± 22.31), respectively, showing a significant difference between the two groups (t = 12.297, p = < 0.001* *). Based on the results of the paired- t-test, the score of sustainability consciousness reveals there was a significant difference between pre and post-intervention groups (t = 11.753, P = < 0.001* *) while there was no significant difference between pre and post-test of the control group (t = 1.849, *P* = 0.070).

Furthermore, for the sustainability consciousness subscale: the mean scores of sustainability knowingness, sustainability attitudes, and sustainability behavior of the intervention and control groups at preprogram were (21.23 ± 9.401 and 19.59 ± 8.449), (22.91 ± 9.485 and 21.96 ± 9.371) and (22.88 ± 8.76 and 22.62 ± 8.33) respectively, showing no significant difference between the two groups at (*P* > 0.05). The mean scores of sustainability knowingness, sustainability attitudes, and sustainability behavior of the intervention and control groups of post-program were (35.48 ± 5.837and 20.36 ± 7.276), (31.51 ± 8.23 and 22.16.51 ± 8.47) and (32.65 ± 6.256 and 21.78 ± 6.932), respectively, showing there was significant difference between the two groups at (*p* = < 0.001* *).

**Table 2:** Comparison of participants nurse’s sustainability consciousness pre and post-educational program (*n* = 114)

Variables	Pre	Post	ES*(Cohen’s d)	T-test	*P*
	Mean ± SD	Mean ± SD			
• **Sustainability Consciousness**					
Intervention	68.35 ± 14.55	103.76 ± 21.81	27.62928	11.753	< 0.001* *
Control	70.01 ± 13.34	71.63 ± 22.31	1.531	1.849	0.070
Independent t-test (*P*-value)	1.291(0.200)	12.297(< 0.001* *)			
• **Sustainability knowingness**					
Intervention	21.23 ± 9.401	35.48 ± 5.837	11.55408	12.240	< 0.001* *
Control	19.59 ± 8.449	20.36 ± 7.276	1.264	0.314	0.754
Independent t-test (*P*-value)	0.030(0.976)	9.631(< 0.001* *)			
• **Sustainability attitudes**					
Intervention	22.91 ± 9.485	31.51 ± 8.23	12.47484	6.509	< 0.001* *
Control	21.96 ± 9.371	22.16.51 ± 8.47	1.822	1.539	0.129
Independent t-test (*P*-value)	1.742(0.365)	3.676(< 0.001* *)			
• **Sustainability behavior**					
Intervention	22.88 ± 8.76	32.65 ± 6.256	10.85955	9.608	< 0.001* *
Control	22.62 ± 8.33	21.78 ± 6.932	1.093	2.612	0.076
Independent t-test (*P*-value)	1.537(0.127)	6.177(< 0.001* *)			

** highly statistically significant at *p* < 0.001

Table 3 clarifies the baseline for green advocacy and green behavior intention, the mean scores of green advocacy, in the intervention and control groups at pre-program were (7.26 ± 2.65and 8.19 ± 2.35), respectively, showing no significant difference between the two groups at (*P* > 0.05). The mean scores of green advocacy in the intervention and control groups were (11.50 ± 2.057and 8.16 ± 2.37), respectively the program, showing there were significant differences between the two groups (t = 7.315, *p* = < 0.001* *). Based on the results of the paired- t-test, the scores of green advocacy there was a significant difference between pre and post-intervention groups (t = 10.965, *P* = < 0.001* *), while there was no significant difference between pre and post-test of the control group groups (t = 1.204, *P* = 0.692) one-month post-program.

**Table 3:** Comparison of participants nurse’s green advocacy and green behavioral intention pre and post-educational program (*n* = 114)

Variables	Pre	Post	ES*(Cohen’s d)	T-test	*P*
	Mean ± SD	Mean ± SD			
**Green Advocacy**					
Intervention	7.26 ± 2.65	11.50 ± 2.057	3.15191	10.965	< 0.001* *
Control	8.19 ± 2.35	8.16 ± 2.37	0.926	1.204	0.692
Independent t-test (P-value)	1.346(0.181)	7.315(< 0.001* *)			
**Green behavioral intention**					
Intervention	6.210 ± 2.130	12.315 ± 2.366	3.06094	14.320	< 0.001* *
Control	6.89 ± 2.94	7.08 ± 2.665	1.826	1.365	0.394
Independent t-test (P-value)	1.684(0.095)	8.274(< 0.001* *)			

** highly statistically significant at *p* < 0.001

Concerning green behavioral intention, the mean scores of green advocacy, in the intervention and control groups at pre-grogram were (6.210 ± 2.130 and 6.89 ± 2.94), respectively, showing no significant difference between the two groups at (*P* > 0.05). The mean scores of green behavioral intention in the and control groups were (12.315 ± 2.366 and 7.08 ± 2.665), respectively after the grogram, showing there were significant differences between the two groups (t = 8.274, p = < 0.001* *). Based on the results of the paired- t intervention test, the scores of sustainability consciousness there was a significant difference between pre and post-intervention groups (t = 14.320, *P* = 0.000) while there was no significant difference between pre and post-control group groups (t = 1.365, *P* = 0.394) one month after the program.

**Fig. 2:**
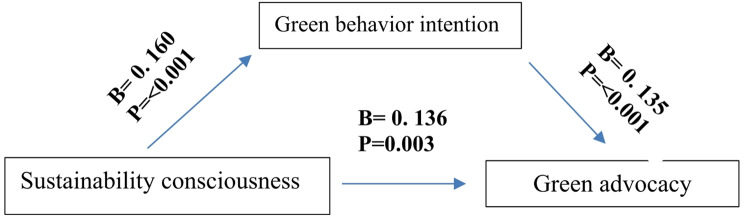
Mediating model of the effect of green behavioral intention between sustainability consciousness and green advocacy at the evaluation phase among the intervention group

Table 4 & Fig. 2 reveals that there was a significant direct effect of sustainability consciousness on green advocacy and green behavioral intention with (B = 0.136, t = 2.117, *P* = 0.003) and (B = 0. 160, t = 5.098, P = < 0.001* *). Also, there was a significant direct effect of green behavioral intention on green advocacy (B = 0.135, t = 6.817, P = < 0.001* *). Regarding the indirect effect between sustainability consciousness and green advocacy, results showed that green behavioral intention plays a partial mediation role between sustainability consciousness and green advocacy that had a statistically significant relation with (B = 0.221, t = 4.491, P = < 0.001* *).

**Table 4:** Mediating effect of green behavioral intention between sustainability consciousness and green advocacy at the evaluation phase among the intervention group

	(B)	CI 95%	t	*p*
**Direct effect**				
Sustainability consciousness → green advocacy	0.136	(0.175–0.377)	2.117	0.003*
Sustainability Consciousness → Green behavioral intention	0. 160	(0.542–0.771)	5.098	< 0.001* *
Green behavioral intention → Green advocacy	0.135	0.163–0.507)	6.817	< 0.001* *
**Indirect effect**				
Sustainability Consciousness → Green behavioral intention → Green advocacy	0.221	(0.221-0.10)	4.491	< 0.001* *

*R* = 0.518, R Square = 0.269, F = 20.419, *P* < 0.001

## Discussion

The damage that global climate change is causing to our world is growing. It’s time to confront this environmental issue head-on, along with the social and economic ones. It becomes necessary to raise public understanding of the significance of sustainable development from a collective responsibility perspective [36]. Adopting more virtuous actions is a challenge for everyone, adults and youth alike, to achieve a brighter future. One of the larger sectors that uses a lot of food, water, plastics, and energy is the health systems industry [2]. High-quality care can be delivered while lowering emissions, waste, and plastic creation through the implementation of climate-friendly initiatives and sustainable awareness [1].

The present study aimed to enhance nurses’ sustainability consciousness and assess its effect on green advocacy and green behavior intention through educational programs. The results indicated that the total participants’ mean score of sustainability consciousness and its subscale had significantly improved among participant nurses of the intervention group between the pre and post-program phases. This could be explained by the fact that green education is essential to fostering sustainable development because it gives people the information, abilities, attitudes, and values they need to confront environmental issues and work toward a more sustainable future. Green education, which aims to increase knowledge and comprehension, brings environmental problems like pollution, resource depletion, biodiversity loss, and climate change to people’s attention. It assists people with comprehending the origins, effects, and connections between these difficulties, which promotes healthy behavioral adjustments and the development of habits.

Prior research has elucidated the function of training programs in enhancing knowledge, conduct, and disposition. People with knowledge are more inclined to take part in community projects, push for legislative reforms, and patronize ecologically conscious companies—all of which are actions that advance sustainability [37]. Employees carry out organizational efforts and can have an impact if they understand the significance of an action and how to carry it out. Staff conduct is influenced by training, which has a significant impact on its duration, intensity, and zeal [38]. Also, a Pakistani study reported that Education for sustainable development in Pakistani higher education institutions had a high level of improved participants’ practices and attitudes toward climate change [39] Other studies supported that sustainable practices had a direct impact on sustainable performance moderating the role of supply chain visibility [40].

On the one hand, “green” training benefits businesses (using best practices and improving environmental performance, for example) as well as workers (encouraging pro-environmental conduct through individual recognition) [41]. According to a recent study, nurses’ awareness of sustainability was lacking [42]. Given that both groups’ staff nurses had low levels of sustainability consciousness before the training, this validates the findings of the current study. Algaber et al. (2023) [43] Discovered that merely 50% of the nurses under investigation had a low degree of sustainability consciousness.

In line with Lester et al. (2022) [44], who demonstrated that education for sustainable development—which aims to create a society with sustainable living behaviors—improves participants’ sustainability consciousness, receiving education enhances participants’ sustainability consciousness after the training program.

The study’s conclusions showed that, when compared to the pre-program exam, the participants’ intentions for green behavior and their advocacy for green issues among nurses had significantly improved by the end of the program. Another earlier Chinese study [45], that found a correlation between educational achievement and higher levels of intention and practice for green actions supports this conclusion. Additionally, Yusoh et al. (2022) [46], proposed that a green training program be included as a human resources activity to enhance the implementation of green practice activities and behavior intention. According to Yusoh’s (2022) exploratory descriptive study conducted in Egypt, most participants exhibited insufficient levels of green practices and behavior intention. It also corroborated the study’s findings that the control and intervention groups had low pre-program test intentions for green activity [47].

According to Luque-Alcaraz et al. (2024) [42], who examined 314 staff nurses drawn from Spanish public and private institutions, nurses with greater awareness of sustainability were more likely to practice eco-friendly behaviors like cutting back on waste, saving energy, and making eco-friendly purchases (*p* < 0.05). It is suggested that green practice (GP) could be a significant result of the environmental training program based on the social exchange idea. Training is defined as a crucial HRM procedure that helps people develop the attitudes, abilities, and ideas that lead to better performance at work [41, 48]Conducted a study in the Italian healthcare industry and found that green training programs encouraged employees to adopt green behavior and had a lasting impact on them. However, several earlier research [47, 49–51] shown that schooling was a more effective means of fostering green practices than training initiatives. In order to inspire people to adopt sustainable practices in their daily lives, green education seeks to cultivate in students a profound appreciation for the environment and its inhabitants as well as a sense of empathy and closeness with the natural world.

According to the study’s findings, which were based on the viewpoints of nurses who participated in a post-education program intervention group, green behavioral intention significantly mediates the association between sustainability consciousness and green advocacy. The reason for this could be that in order to be more dedicated to eco-friendly procedures and everyday activities, nurses must demonstrate high levels of green intention behavior. Therefore, through seminars and instruction on sustainability consciousness, researchers, educators, and companies must engage present employees in enhancing their green intended behavior.

Wu and Chiang (2023) [52] Provided support for this study’s findings by reporting a significant relationship between environmental awareness, green advocacy, green self-efficacy, and green word-of-mouth intention. In addition, they showed that people with high environmental awareness levels are more receptive to ethical practices like recycling and buying green products, as well as more open to and agreeable to environmentally friendly principles.

Along the same line, Cheng et al., (2022) [24] explored that not only sustainability awareness but employees who believe in their environmental accountability and have high green intentions are more likely to encourage coworkers to behave pro-environmentally and become green advocates within organizations. Furthermore, Santos., & Ramirez, (2022) [51] revealed that when employees who have more knowledge and are highly oriented about sustainability and climate change, they have high green behavior intentions and participate adequately in green advocacy practices, Also they indicated that green behavioral intentions had the mediating effect on the relationship between environmentally knowledgeable awareness and green and advocating behavior.

This result aligns with the planned behavior idea. People behave the way they do because of their intentions, which are shaped by their attitudes [20]. This outcome is in line with the findings of Li et al. (2021) [53], who discovered a favorable correlation between behavioral intentions and associated practice. Nurses’ intentions to conduct green behavior have a favorable impact on their green practices. The resolve to act sustainably has a favorable impact on nurses’ work as green advocates [34]. This finding is consistent with past studies on the subject, including one by Miaomiao et al. (2021) [53], who discovered a link between related practice and behavioral intentions. The intention-behavior link has also shown the moderating function of corporate green education support toward green practices [54].

## Conclusion

Raising nurses’s awareness regarding the sustainable facts on nursing practices and its relation to eco-friendly environment can provoke their green behavior intention and direct them to be green advocator. Furthermore, A high level of green intention maximizes the effect of sustainability consciousness on developing green advocacy.

### Implications for nursing education and practices

Theoretical implication: the study adds to the existing body of nursing knowledge. Also, The study is significant as it establishes the relationship between sustainability consciousness, green advocacy, and green behavior intention, which is very necessary to ensure the sustainability of the hospitals to be more efficient in the use of resources.

Social implications: The study’s findings are crucial in explaining how increasing sustainability consciousness will not only impact the hospital staff and its management but also benefit society at large. The study proves that the adoption of SC will raise a generation of nurses who are sufficiently aware of environmentally sustainable actions. The crucial findings may be adopted by policymakers to ensure incorporating education courses for undergraduate nursing students on sustainability consciousness, green intention, and green advocacy.

Practical implications: To raise a generation of nurses who are sufficiently aware of environmentally sustainable actions, researchers suggested incorporating education courses for undergraduate nursing students on sustainability consciousness, green intention, and green advocacy. Along the same line, Hospital managers should set strategies to raise healthcare providers’ awareness of sustainability issues and environmentally friendly practices. In addition, hospital administrations need to introduce green duties and sustainability policies to encourage employees to develop green behavior intention, and with reinforcement strategies, they will become green advocators. Future studies should be conducted involving multiple sites or international samples to confirm the findings.

### Limitations of the study

The scope of the study was limited to a single hospital. As the study shows a positive relationship between the variables, future studies can be conducted on a larger sample and multiple hospital settings. The scope of the study was also limited to the three variables; as such, further studies can be undertaken to analyze the impact of external variables such as existing hospital policies, prior environmental initiatives, or other educational programs running concurrently that might influence the outcomes. Additionally, the study included nurses, so further studies should be applied to other health professions.

## Data Availability

No datasets were generated or analysed during the current study.

## Abbreviations

SC: Sustainability consciousness
BI: Behavioral intention, UNESCO’s: United Nations Educational, scientific and cultural organization
UNESCO’s: United Nations Educational, scientific and cultural organization
SPSS: Statistical package for social sciences
GP: Green practice
HRM: Human resources management
